# CK2α, over-expressed in human malignant pleural mesothelioma, regulates the Hedgehog signaling pathway in mesothelioma cells

**DOI:** 10.1186/s13046-014-0093-6

**Published:** 2014-11-25

**Authors:** Shulin Zhang, Yi-Lin Yang, Yucheng Wang, Bin You, Yuyuan Dai, Geraldine Chan, David Hsieh, Il-Jin Kim, Li Tai Fang, Alfred Au, Hubert J Stoppler, Zhidong Xu, David M Jablons, Liang You

**Affiliations:** Department of Thoracic Surgery, The Fifth Hospital of Dalian, Dalian, 116021 P.R. China; Thoracic Oncology Laboratory, Department of Surgery, Helen Diller Family Comprehensive Cancer Center, University of California San Francisco, San Francisco, CA 94143 USA; Department of Surgery, Helen Diller Family Comprehensive Cancer Center, University of California San Francisco, San Francisco, CA 94143 USA; Department of Thoracic Surgery, Beijing Chao-Yang Hospital, Affiliated with Capital University of Medical Science, Beijing, P.R. China; Division of Diagnostic Pathology, Helen Diller Family Comprehensive Cancer Center, University of California San Francisco, San Francisco, CA 94143 USA; Tissue Core, Comprehensive Cancer Center, University of California San Francisco, San Francisco, CA 94143 USA

**Keywords:** CK2α, Hedgehog, Gli1, IHC, Mesothelioma

## Abstract

**Background:**

The Hedgehog (Hh) signaling pathway has been implicated in stem cell maintenance and its activation is aberrant in several types of cancer including mesothelioma. Protein kinase CK2 affects several cell signaling pathways through the mechanism of phosphorylation.

**Methods:**

Protein and mRNA levels of CK2α and Gli1 were tested by quantitative RT-PCR and immunohistochemistry staining in mesothelioma samples and cell lines. Down-regulated Gli1 expression and transcriptional activity were demonstrated by RT-PCR, Western blot and luciferase reporter assay.

**Results:**

In this study, we show that CK2α is over-expressed and a positive regulator of Hegdehog/Gli1 signaling in human malignant pleural mesothelioma. First of all, we found that the mRNA levels of CK2α and Gli1 were broadly elevated and correlated (n = 52, r = 0.401, P < 0.05), compared with LP9 (a normal mesothelial cell line). We then investigated their expression at the protein level, and found that all the 7 mesothelioma cell lines tested showed positive staining in CK2α and Gli1 immunohistochemistry. Correlation analysis by Pearson test for CK2α and Gli1 expression in the 75 mesothelioma tumors and the 7 mesothelioma cell lines showed that the two protein expression was significantly correlated (n = 82, r = 0.554, P < 0.01). Furthermore, we demonstrated that Gli1 expression and transcriptional activity were down-regulated after CK2α was silenced in two mesothelioma cell lines (H28 and H2052). CK2α siRNA also down-regulated the expression of Hh target genes in these cell lines. Moreover, treatment with a small-molecule CK2α inhibitor CX-4945 led to dose-dependent inhibition of Gli1 expression and transcriptional activity. Conversely, forced over-expression of CK2α resulted in an increase in Gli1 transcriptional activity in H28 cells.

**Conclusions:**

Thus, we report for the first time that over-expressed CK2α positively regulate Hh/Gli1 signaling in human mesothelioma.

**Electronic supplementary material:**

The online version of this article (doi:10.1186/s13046-014-0093-6) contains supplementary material, which is available to authorized users.

## Background

Protein kinase CK2 (formerly known as casein kinase II) is a highly conserved serine/threonine kinase that phosphorylates more than 300 proteins [1]. CK2 has a heterotetrameric structure consisting of two catalytic subunits (42-kDa α or 38-kDa α’) and the regulatory subunit (28-kDa β), forming the configurations α2β2, αα’β2 and α’2β2. CK2 is a multifunctional protein kinase [2], that has been shown to be involved in nearly every aspect of cell proliferation and survival [3-5]. The level of CK2α expression is tightly regulated in normal cells [6], and increased CK2α level and activity has been consistently observed in a variety of human cancers [7-9]. For instance, a high level and/or nuclear localization of CK2α is a marker of poor prognosis for patients with acute myeloid leukemia, chronic lymphocytic leukemia, prostate cancer and gastric cancer [10-13]. In addition, stable knockdown CK2α has recently been shown to inhibit cell migration and invasion of hepatocellular carcinoma [14]. Studies also revealed that CK2 affects several cell signaling pathways, including PI3K, NFkB and Wnt [6,15,16].

The Hedgehog (Hh) family of secreted proteins, which consists of Sonic, Indian and Desert Hedgehog, plays important roles in mammalian development and in stem cell maintenance [17,18]. The Hh pathway is activated at the cell surface by the Hh ligand binding to its receptor Patched (Ptc), resulting in derepression of the effector protein, a G-protein-coupled receptor, Smoothened (Smo) [19]. Ultimately, Smo activates the Gli family of transcription factors and target genes. There are three Gli proteins in humans: Gli1 activates Hh target genes, Gli2 acts both as activator and repressor of Hh target genes, while Gli3 acts as a repressor of Hh target genes [20,21]. Deregulation of Hh/Gli signaling is implicated as an initiating or maintaining factor in the progression of various cancers, including basal cell carcinomas, medulloblastomas, leukemia, gastrointestinal, lung, ovarian, breast and prostate cancers [20,22]. For instance, the Gli1 gene is amplified in human glioma and activated in basal cell carcinoma [23-25]. Transgenic over-expression of Gli1 in mice leads to the development of basal cell carcinoma [26]. Hedgehog pathway inhibitor GDC-0449 is an FDA-approved drug for treatment of metastatic basal cell carcinoma.

Malignant pleural mesothelioma (mesothelioma) is an aggressive cancer with poor prognosis that originates mostly from the pleura of the lung. Mesothelioma is associated with occupational exposure to asbestos [27]. It has been suggested that mesotheliomas contain cancer stem cells and their stem cell characteristics are thought to confer therapy-resistance [28]. Activated stem cell signaling in patients has already been suggested in mesothelioma. For instance, cells staining positive for nuclear beta-catenin, a marker for Wnt signaling activation, have been reported in a few studies [29,30].

Recently, Hh/Gli1 signaling was reported to be aberrantly activated in human mesothelioma [31,32]. To date, there is no evidence for the status of CK2 in mesothelioma. The relationship between CK2 and Hh/Gli1 signaling in mesothelioma is unknown. We therefore sought to detect CK2α and Gli1 expression in primary mesothelioma tissues and cell lines, and to analyze their relationship.

## Methods

### Cell culture and small molecule treatment

Nine human mesothelioma cell lines (H28, H290, H2052, 211H, H2452, MS-1, H226, REN and H513) and a normal pleura cell line (LP9) were obtained from American Type Culture Collections (Manassas, VA). Cells were routinely maintained in RPMI-1640 supplemented with 10% heat-inactivated fetal bovine serum, penicillin (100 μg/ml) and streptomycin (100 μg/ml). All cells were routinely cultivated at 37°C in a humid incubator with 5% CO2. Treatment with CX-4945 (Synkinase, San Diego, CA) and TBB (Sigma, St. Louis, MO) dissolved in DMSO was administered at several dosages. Cells were grown in medium for 48 hours after treatment. Cell proliferation *in vitro* was assessed using a CellTiter-Glo Luminescent cell viability assay (Promega Corporation, Madison, WI), according to the manufacturer’s protocol [33].

### Tissue samples and immunohistochemistry (IHC)

Fresh mesothelioma tissues were obtained from patients who were undergoing surgical resection of the primary tumor. All human tissue samples were obtained and analyzed in accordance with procedures approved by the institutional review board of the University of California, San Francisco (IRB H8714-22 942–01). The tissue microarray sections were immunostained as previously described [33]. Anti-CK2α antibody was from Millipore (Billerica, MA). Anti-Gli1 antibody was from Cell Signaling (Beverly, MA). The following scoring system was used: −, no stain; +, weak staining (10% or above stained cellularity considered as positive); ++, moderate staining (30% or above stained cellularity considered as positive); +++, strong staining (50% or above stained cellularity considered as positive). All scoring was done under low power objective lens (10×) with a Zeiss Axioscop 2 microscope (Carl Zeiss Inc, Germany). Images were taken under 10x or 40x objective lens.

### SiRNA and plasmid DNA transfection

CK2α siRNA (ON-TARGET plus SMARTpool) and control siRNA were purchased from Thermo Scientific (Waltham, MA). Cells were seeded in a 6-well plate as 10^5^ cells/well one day before transfection, with a target of 30-50% confluency at the time of transfection. Cells were transfected with 50 nmol/L of siRNA using Lipofectamine RNAiMAX (Invitrogen, Carlsbad, CA) according to the manufacturer’s protocol. Adequate inhibition of the siRNA-mediated knockdown was confirmed by Western blot. The pcDNA3.1-CK2α or control pcDNA3.1-LacZ plasmid vectors were then transfected into the H28 cells (0.5 μg/ml in 24-well plate) using Lipofectamine 2000 transfection reagent (Invitrogen), according to the manufacturer’s protocol. Cells were harvested for RT-PCR and Western blot or used in reporter assays at 48 hours post-transfection.

### RNA isolation, cDNA synthesis and semi-quantitative RT-PCR

RNA was isolated using the RNeasy Mini kit (Qiagen, Valencia, CA). Human Lung Total RNA was purchased from Applied Biosystems (Foster City, CA). Five-hundred ng of total RNA was converted into 20 μl cDNA using iScript cDNA Synthesis Kits (Bio-Rad, Hercules, CA,) according to the manufacturer’s recommendations. PCR bands were visualized under UV light and photographed.

### Quantitative RT-PCR

A total of 2 μl of the reverse transcription reaction were used as template for real-time detection using TaqMan Technology on an Applied Biosystems 7000 sequence detection system (Applied Biosystems). Gene expression was quantified for the tested genes and endogenous control gene b-glucuronidase (*GUSB*) using commercially available primer and probe sequences (Applied Biosystems). Relative mRNA level was calculated by normalizing the gene expression levels of tested genes to that of the control *GUSB* gene.

### Western blot analysis

Whole protein was extracted by M-PER Mammalian Protein Extraction Reagent (Thermo) from cell lines added with Phosphatase Inhibitor Cocktail Set II (Calbiochem, San Diego, CA) and Complete Protease Inhibitor Cocktails (Roche, Lewes, UK) according to manufacturers’ protocols. The proteins were separated on 4–15% gradient SDS–polyacrylamide gels and transferred to Immobilon-P membranes (Millipore, Bellerica, MA). The following primary antibodies were used: anti-CK2α (Millipore), anti-Gli1 (Cell Signaling), and anti-GAPDH (Trevigen, Gaithersburg, MD). After being incubated with appropriate secondary antibodies, the antigen-antibody complexes were detected by using an ECL blotting analysis system (Amersham Pharmacia Biotech, Piscataway, NJ). Digital images were prepared using Adobe Photoshop CS3.

### Luciferase reporter assays

To measure Gli-mediated Hh transcriptional activity, the luciferase reporter constructs, 8× wild-type Gli binding site (8× Gli ^wt^ Luc) or 8× mutant Gli binding site (8× Gli ^mut^ Luc) plasmids [34] and a human Gli1 expression vector (pcDNA3.1-Gli1) were co-transfected into H28 cells in 24-well plates. The Renilla luciferase pRL-TK plasmid (Promega, Madison, WI), whose expression is driven by the housekeeping thymidine kinase gene promoter, was co-transfected to normalize for transfection efficiency. All transfection experiments were performed using the Lipofectamine2000 (Invitrogen) in accordance with the manufacturer’s instructions. After 24 h cells were lysed and luciferase assays were performed as described previously [35]. Results are expressed as fold induction, which is the ratio of luciferase activity induced in Gli-transfected cells relative to basal luciferase activity in control transfected H28 cells. All experiments were performed in triplicate; means and standard errors were calculated using Student’s t-test.

### Statistical analysis

Data were expressed as mean ± standard deviation (SD) from three independent experiments. All of the statistical analyses were performed using SPSS 13.0 for Windows software system (SPSS Inc, Chicago, IL). Student’s t-test was used to compare the differences among groups. Pearson product correlation tests (in the form of a correlation matrix) were used to analyze the mRNA and protein level of CK2α and Gli1. A significant difference was declared if the P value from a two-tailed test was less than 0.05 (* P <0.05, ** P <0.01).

## Results

### Quantitative RT-PCR of CK2α and Gli1

**Figure 1:**
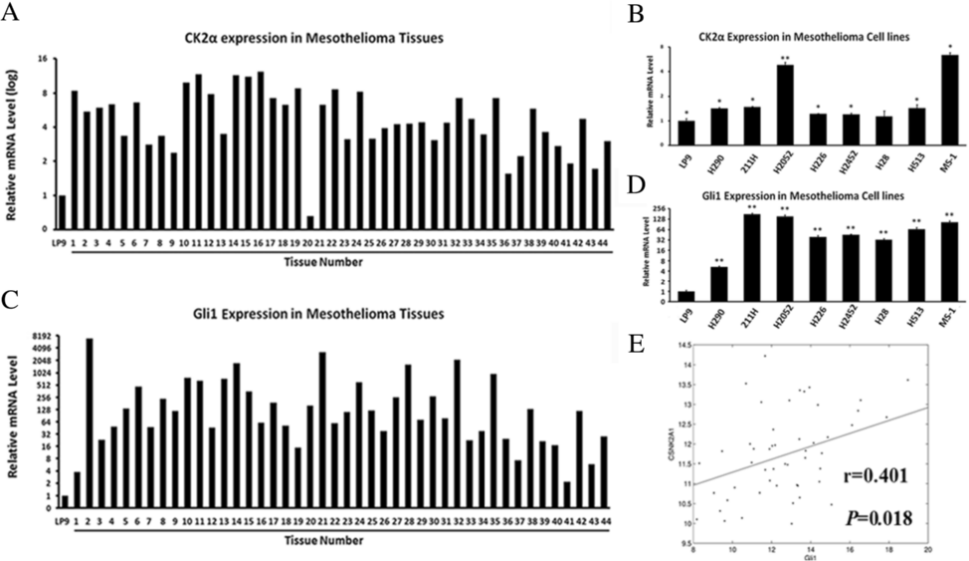
**mRNA level and correlation of CK2α and Gli1. (A)** CK2α in primary mesothelioma samples. **(B)** CK2α in mesothelioma cell lines. **(C)** Gli1 in primary mesothelioma samples. **(D)** Gli1 in mesothelioma cell lines. **(E)** Correlation between CK2α and Gli1 mRNA levels (r = 0.401, P < 0.05).

Quantitative RT-PCR was used to examine the mRNA level of CK2α and Gli1 in 44 primary mesothelioma samples and 8 mesothelioma cell lines (H28, H290, H2052, 211H, H2452, MS-1, H226 and H513). As shown in Figure 1A, 97.7% (43/44) primary samples showed dramatically elevated CK2α mRNA level, compared with a normal pleura cell line LP9. In analysis of the cell lines, 87.5% (7/8) showed significantly elevated CK2α expression mRNA level, compared with LP9 (Figure 1B). All of the primary samples and the cell lines showed elevated Gli1 mRNA level, compared with LP9 (Figure 1C-D).

### Immunohistochemistry of CK2α and Gli1 in mesothelioma

**Figure 2:**
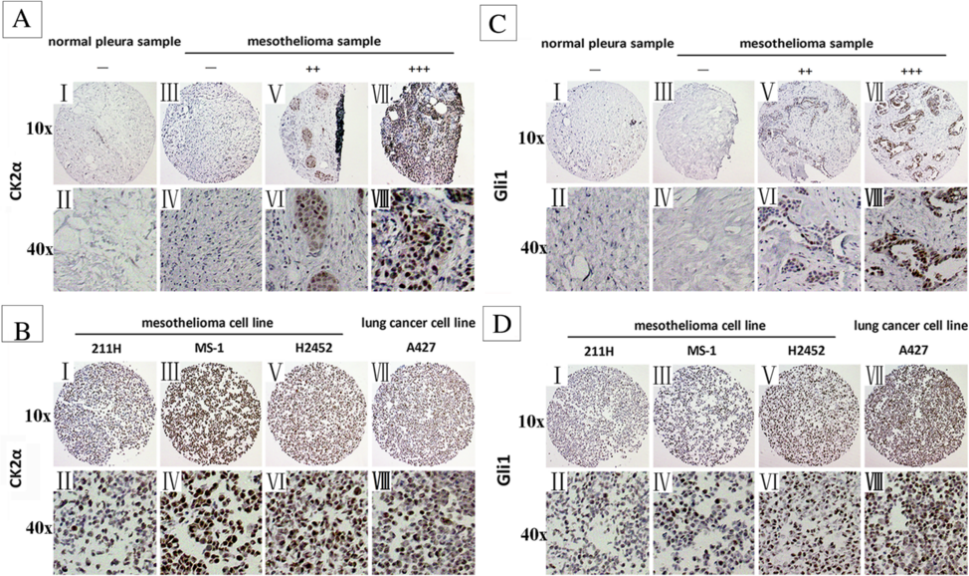
**IHC of CK2α and Gli1 in primary mesothelioma samples and cell lines. (A)** CK2α in primary mesothelioma samples. (I, II) normal pleura sample; (III– VII) mesothelioma samples. **(B)** CK2α in mesothelioma cell lines. (I, II) 211H, ++; (III, IV) MS-1, +++; (V, VI) H2452, +++; (VII, VII) Lung cancer cell line A427 (positive control), +++. **(C)** Gli1 in primary mesothelioma samples. (I, II) normal pleura sample; (III– VII) mesothelioma samples. **(D)** Gli1 in mesothelioma cell lines. (I, II) 211H, ++; (III, IV) MS-1, +++; (V, VI) H2452, +++; (VII, VII) Lung cancer cell line A427 (positive control), +++. Images were taken under 10x or 40x objective lens.

Both CK2α and Gli1 genes are over-expressed in a variety of cancers [36,37]. However, whether CK2α is over-expressed in mesothelioma is unknown. First, by using the immunohistochemistry (IHC) staining, we detected the protein level of CK2α and Gli1 in 75 primary mesothelioma samples and 7 cell lines (H28, H290, 211H, H2452, MS-1, H226 and REN) of the tissue microarray sections. The positive or negative results of CK2α and Gli1 staining in these sections are shown in Figure 2 and Tables 1, 2 and 3. Lung cancer cell line A427 with strong positive (+++) staining of CK2α and Gli1 was used as a positive control. In the primary mesothelioma samples (Figure 2A, C and Tables 1 and 2), the overall positive ratios of CK2α and Gli1 were both 93.3% (70/75). The moderate and strong positive (++/+++) ratio was 69.3% (52/75) in CK2α staining and 50.6% (38/75) in Gli1 staining. The strong positive (+++) ratio was high (40%) in CK2α staining. The strong positive ratio of Gli1 staining was lower (17.3%) than that of CK2α staining. In specimens of normal pleural tissue, CK2 and Gli1 staining was negative (−) or weak (+) at the cell membrane or in the region of cytoplasm near the membrane (Figure 2A, C and Tables 1 and 3). In all the 7 cell lines tested, both CK2α and Gli1 showed positive staining (Figure 2B, D and Table 1). For CK2α staining, H290, H2452 and MS-1 were strong positive (+++), while REN and H226 were weaker (+). For Gli1 staining, H290, H2452 and MS-1 were strong positive (+++), while H28 was weaker (+). These results suggest an aberrant over-expression of CK2 and Gli1 in mesothelioma.

**Table 1:** **Immunohistochemistry findings from human mesothelioma samples**

**Sample no.**	**IHC of anti-CK2α**	**IHC of anti-Gli1**	**Sample no**	**IHC of anti-CK2α**	**IHC of anti-Gli1**
T01	-	+	T45	+	+
T02	+	++	T46	+++	+++
T03	++	+	T47	+++	+
T04	+	+	T48	+	+
T05	+	+	T49	++	++
T06	++	++	T50	+++	++
T07	+++	++	T51	+++	++
T08	++	+	T52	+++	-
T09	+	++	T53	+++	++
T10	++	++	T54	++	+
T11	+++	+++	T55	+++	+
T12	+++	++	T56	++	+
T13	+++	+	T57	++	++
T14	+	+	T58	+++	+++
T15	+++	++	T59	++	+++
T16	++	++	T60	-	++
T17	++	+	T61	+	+
T18	+	+	T62	++	++
T19	++	+	T63	+++	+
T20	+++	+++	T64	+++	++
T21	+++	+++	T65	++	+++
T22	-	-	T66	++	+
T23	+++	++	T67	+++	+++
T24	+	+++	T68	+++	+++
T25	+++	++	T69	+	+
T26	+++	+++	T70	+	+
T27	++	++	T71	+++	+++
T28	+++	+	T72	+++	+
T29	+++	++	T73	+++	+
T30	++	+	T74	-	-
T31	+++	++	T75	+	++
T32	++	+	N1	-	-
T33	+	-	N2	+	+
T34	+	+	N3	+	-
T35	+	+	N4	-	-
T36	+	+	N5	-	-
T37	++	+	N6	-	-
T38	-	-	H226	+	++
T39	+++	++	REN	+	++
T40	+++	++	H290	+++	+++
T41	++	+	211H	++	++
T42	++	++	H2452	+++	+++
T43	+	+++	H28	++	+
T44	++	+	MS-1	+++	+++

N = normal tissue; T = tumour tissue; IHC = immunohistochemistry; − = no stain; + = weak stain; ++ = moderate stain; +++ = strong stain

.

**Table 2:** **Positive and negative number and ratio of CK2α and Gli1 in 75 primary mesothelioma samples**

	**− Number (ratio)**	**+ Number (ratio)**	**++ Number (ratio)**	**+++Number (ratio)**	**Total (ratio)**
CK2α	5 (6.67%)	18 (24.0%)	22 (29.3%)	30 (40.0%)	75 (100%)
Gli1	5 (6.67%)	32 (42.7%)	25 (33.3%)	13 (17.3%)	75 (100%)

**Table 3:** **Positive and negative number of CK2α and Gli1 in normal pleura samples**

	**− Number**	**+ Number**	**++ Number**	**+++Number**	**Total**
CK2α	4	2	0	0	6
Gli1	5	1	0	0	6

### Correlation between CK2α and Gli1

To analyze the relationship between CK2α and Gli1 in mesothelioma, we performed a Pearson product correlation test on CK2α and Gli1 staining in the 75 primary samples and the 7 cell lines. CK2α and Gli1 were mildly correlated at the protein level (n = 82, r = 0.554, P < 0.01, Table 4). We then investigated their relationship at the mRNA level, and found that CK2α and Gli1 were also broadly elevated and correlated (n = 52, r = 0.401, P < 0.05, Figure 1E and Table 4) at the mRNA level.

**Table 4:** **Correlation analysis of CK2α and Gli1 in mesothelioma**

	**N**	**r**	***P***
mRNA	52	0.401	0.018
Protein	82	0.554	0.000

### Down-regulation of Hh/Gli1 signaling by CK2α knockdown

**Figure 3:**
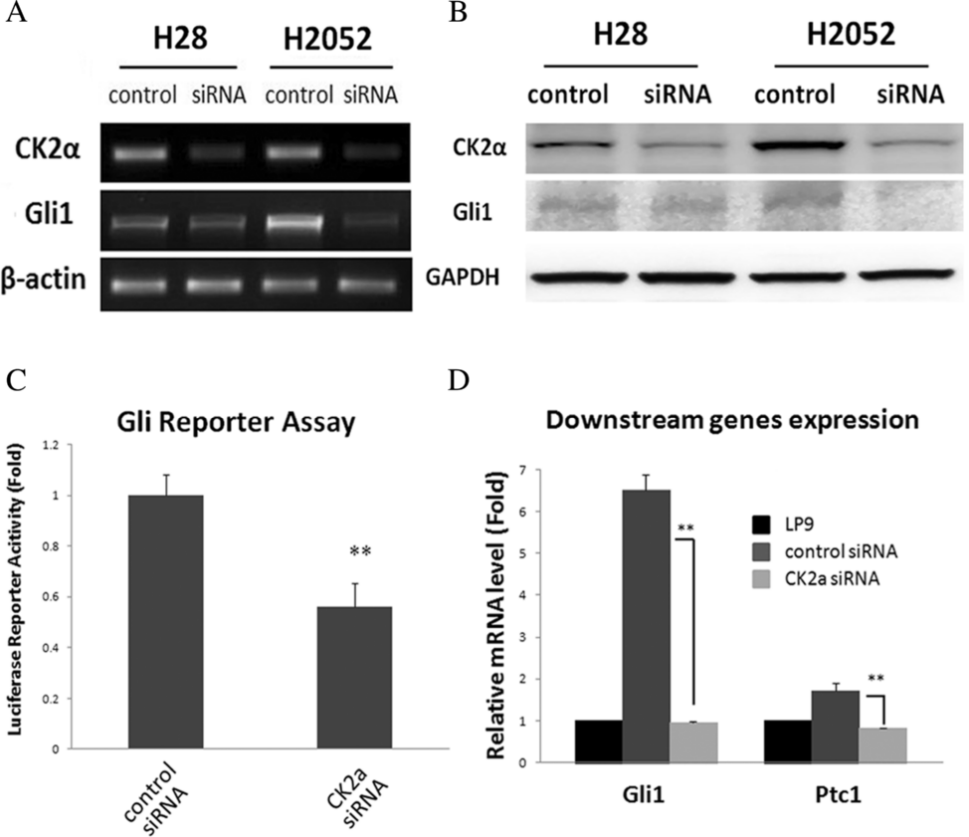
**Down-regulation of Gli1 expression and transcriptional activity after CK2α knockdown in mesothelioma cell lines. (A)** Gli1 mRNA levels after treatment with CK2α siRNA. **(B)** Gli1 protein levels after treatment with CK2α siRNA. **(C)** The transcriptional activity of Gli1 after treatment with CK2α siRNA in H28. Silencing of CK2α resulted in a significant decrease (more than 48% at 50 μM siRNA) of the transcriptional activity. **(D)** Down-regulation of two target genes of the Hh pathway (Gli1 and Ptc1), detected by quantitative RT- PCR.

To investigate whether CK2 has an effect on the Hh pathway, we silenced CK2α expression for 48 h using CK2α siRNA in H28 and H2052 cell lines. It has been previously shown that 48 h of CK2α siRNA treatment is efficient to knockdown CK2α protein expression in the treated cells [38-41]. The efficiency of RNA interference was monitored by semi-quantitative RT-PCR and Western blot (Figure 3A and B). The corresponding mRNA and protein levels of CK2α in H28 and H2052 cell lines decreased dramatically (Figure 3A and B, upper lane). Expression of Gli11 was inhibited after CK2α knockdown in the two cell lines (Figure 3A and B, middle lane).

To confirm whether CK2α positively regulates Hh/Gli1 signaling, we performed a luciferase reporter assay to detect the transcriptional activity of the pathway. Silencing of CK2α in H28 cells resulted in a significant decrease (48% at 50 μM, P < 0.01, Figure 3C) in the Gli reporter activity, compared with the non-targeting siRNA (control). To study the effects of CK2α knockdown on Hh/Gli1 signaling, quantitative RT-PCR was performed to compare the levels of Gli1 and Ptc1 mRNA between wild-type and CK2α knockdown cell lines. The mRNA level of the two Hh target genes decreased significantly (P < 0.01, Figure 3D) after CK2α knockdown. In normal mesothelial LP9 cells, decrease in mRNA level of the two Hh target genes was also detected using quantitative RT-PCR (Additional file 1: Figure S1A). These results suggest a depressed transcriptional activity of Hh/Gli1 signaling after CK2α knockdown.

### Down-regulation of Gli1 expression and transcriptional activity by CK2α inhibitor

**Figure 4:**
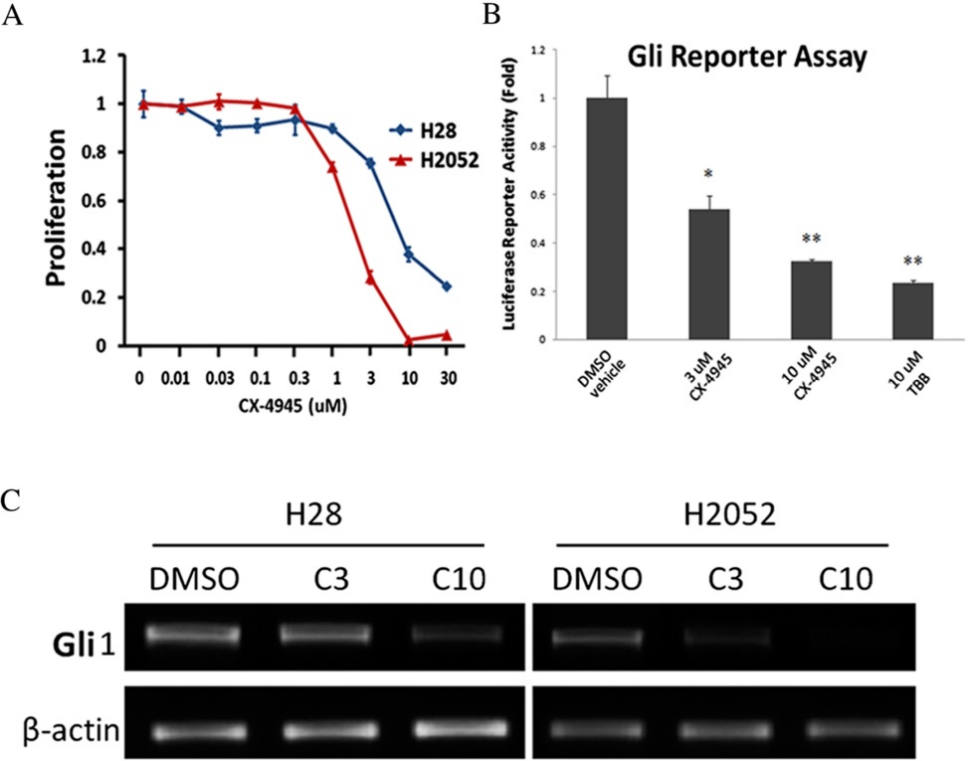
**Down-regulation of Gli1 expression and transcriptional activity after treatment with CX-4945. (A)** Cell proliferation assay after treatment with CX-4945. **(B)** Dose-dependent decrease in Gli reporter activity in H28. The decrease was 47% in the presence of 3 μM CX-4945 or 70% in the presence of 10 μM CX-4945. TBB was used as a positive control. **(C)** Dose-dependent decrease of Gli1 mRNA after treatment with CX-4945. Gli1 expression decreased noticeably at the dosage levels of 3 μM in H2052 or 10 μM in H28, and decreased to minimal at 10 μM in H2052.

To extend our findings to clinical applications, we used a small-molecule CX-4945 (5-(3-chlorophenylamino)benzo[c][2,6]naphthyridine-8-carboxylic acid), a first-in-class, selective, oral inhibitor of CK2α under investigation in Phase 1 clinical trials [42]. Cells were treated with multiple concentrations of CX-4945 (0.01, 0.03, 0.1, 0.3, 1, 3, 10 and 30 μM), or with the vehicle DMSO for 72 hours. The cell proliferation assay demonstrated that treatments with CX-4945 led to cell growth inhibition in a dose-dependent manner, both in H28 and H2052 cell lines (IC50 values were 7.2 μM in H28 and 2.0 μM in H2052, respectively, Figure 4A). Normal mesothelial LP9 cells also showed a dose-dependent inhibition on cell growth by CX-4945 treatment (IC50 values is 3.3 μM, Additional file 1: Figure S1B). After treatment with CX-4945, Gli1 expression decreased noticeably at the dosage level of 3 μM in H2052 or 10 μM in H28, and decreased to minimal at 10 μM in H2052 (Figure 4C). We further demonstrated that treatment with CX-4945 led to a dose-dependent decrease in Gli reporter activity in the H28 cells. The decrease was 47% (P < 0.05) in the presence of 3 μM CX-4945 or 70% (P < 0.01) in the presence of 10 μM CX-4945 (Figure 4B). TBB (4,5,6,7-tetrabromobenzotriazole), a well-known inhibitor of CK2α [43] was used as a positive control. 10 μM TBB led to an 80% decrease of Gli1 transcriptional activity.

### Transcriptional activation of Gli1 by forced over-expression of CK2α

**Figure 5:**
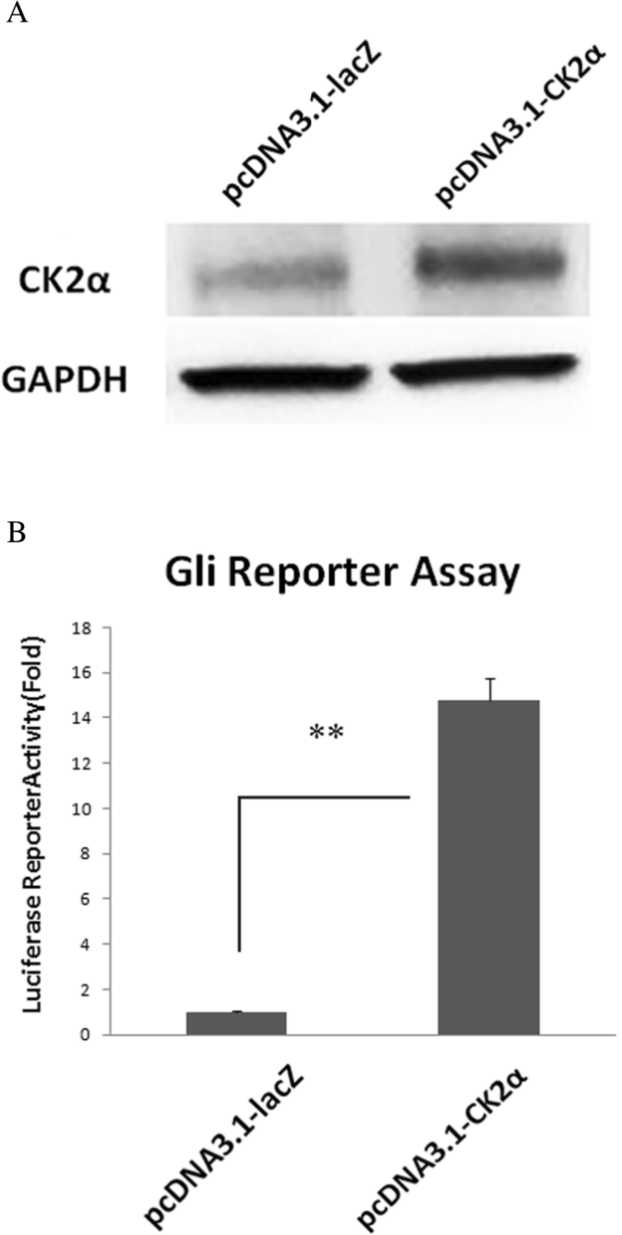
**Up-regulation of Gli1 transcriptional activity in forced over-expressed CK2α H28 cells. (A)** Over-expression of CK2α detected by Western blot. **(B)** Gli1 reporter assay. A significant (>10-fold) increase of the Gli1 transcriptional activity was shown in forced over-expressed CK2α H28 cells.

To confirm whether CK2 positively affects the transcriptional activity of Gli1, we transfected H28 cells with either a pcDNA3.1-CK2α or control pcDNA3.1-LacZ plasmid. As expected, CK2α over-expression, detected by Western blot (Figure 5A), was attributed to the activation of Gli1 in H28 cells. The reporter assay also showed a significant (>10-fold, P < 0.01) increase of Gli1 transcriptional activity (Figure 5B). These findings suggest that forced over-expression of CK2α leads to a transcriptional activation of Gli1.

## Discussion

Our results suggest that CK2α is over-expressed and positively regulates Hh/Gli1 signaling in human malignant pleura mesothelioma. This is supported by several lines of evidence. First, broadly aberrant over-expression of CK2 was demonstrated in most of the primary mesothelioma samples and cell lines we tested. Second, we found a statistically mild correlation between CK2 and Gli1 expression both at the mRNA and protein levels. Third, in experimental studies of mesothelioma cell lines, CK2α inhibition by siRNA or small-molecular inhibitor resulted in down-regulation of Gli1 expression and transcriptional activity. Finally, forced over-expression of CK2α led to Gli1 transcriptional activation.

To date, there is no evidence for the expression status of CK2 in mesothelioma, although CK2 reportedly affects cell growth in several types of tumor. Our study shows the broad over-expression of CK2α for the first time. Our findings suggest that CK2 may play an important role in mesothelioma pathogenesis and is a potential biomarker and therapeutic target for patients with mesothelioma. Further studies are warranted to show evidence for patients who may potentially benefit from CK2 inhibitors. CK2 inhibitors have not been extensively developed as therapeutic agents, partly because the ATP-binding pocket of CK2 is not as druggable as some other protein kinases [44-46]. To date, only one small-molecule CK2 inhibitor has been entered to clinical trials as a potential anticancer drug [47]. CX-4945, a highly selective CK2 small molecule inhibitor, is a promising first-in-class oral therapeutic agent that targets multiple human cancers. CX-4945 shows a favorable safety profile in Phase I clinical trials [48]. In addition, CIGB-300 (a synthetic peptide-based drug targeting the CK2 phosphoaceptor domain) has proved to be safe and of clinical benefit in Phase I cervical cancer trials [49].

Our study also demonstrates that Gli1 is over-expressed in mesothelioma, indicating the presence of an active Hh pathway. In several samples, the Gli1 mRNA level was more than 1000-fold greater than that in the normal pleura cell line LP9. This is consistent with findings from the recent study that first reported the role of aberrant Hh/Gli1 signaling in human mesothelioma [31]. The Hedgehog pathway expression in mesothelioma tumors was recently analyzed using qRT-PCR, and SHH gene expression was only detected in tumor tissue but not in non-tumor pleural samples [31]. Moreover, *in situ* hybridization analysis of mesothelioma tumors showed similar results that the expression of SHH and DHH was mostly associated with the tumor cells [31]. Together, these results suggested that the Hh pathway expression on mesothelioma cells is mostly from autocrine expression of its ligands.

The Hh pathway plays key roles in maintaining cancer stem cells, and an antagonist of hedgehog inhibits tumor growth, indicating a new therapeutic approach on mesothelioma. However the druggable targets in the Hh pathway are very limited; to date, only one Hedgehog inhibitor GDC-0449 is approved by FDA for treatment of metastatic basal cell carcinoma. Recently, we have shown that CK2α regulates Hh/Gli1 signaling in human lung cancer, and silencing of CK2α promotes Gli1 degradation in a time-course experiment [39]. CK2α promotes Hh/Gli1 signaling by, at least in part, increasing Gli1 protein stability [39]. Another study showed that silencing CK2α inhibited the expressions of Gli1 and Patched homolog 1 (PTCH1) in hepatocellular carcinoma, resulting in the inactivation of Hh signaling pathway [14]. The relationship between CK2 and Hh/Gli1 signaling in other types of cancer, including mesothelioma is still unknown.

In this study, correlated IHC staining of CK2α and Gli1 in mesothelioma was demonstrated. For example, 32 of 38 (84.2%) of moderate and strong positive (++/+++) staining of Gli1 existed in the 52 samples with moderate and strong positive (++/+++) staining of CK2α. Nine of thirteen (69.2%) of strong positive (+++) staining of Gli1 exited in the 30 samples with strong positive (+++) staining of CK2α. We also analyzed CK2β expression in human mesothelioma tumors using IHC. The CK2β staining was compared with CK2α and Gli1 expression in the same tumor samples (n = 61) (Additional file 1: Table S1). Association of CK2α and Gli1 expression in the tumors was identified (Additional file 1: Table S2, p < 0.05, chi-square). Association of CK2α and CK2β expression in the tumors was shown (Additional file 1: Table S3, p < 0.05, chi-square). However, association of CK2β and Gli1 expression in the tumors was not statistically significant (Additional file 1: Table S4, p > 0.05, chi-square). Taken together, our results suggested that Gli1 expression is associated with CK2α expression, but not CK2β expression, in the mesothelioma tumors. Furthermore, in the experimental studies of mesothelioma cell lines, CK2α was suggested to positively regulate Hh/Gli1 signaling. Our study also suggests that CK2 is an additional target for the inhibition of Hh/Gli1 signaling in mesothelioma. Thus, CK2 inhibitors such as CX-4945 may be potentially beneficial for clinical treatment of patients with mesothelioma.

## Conclusions

In summary, we report that CK2 is over-expressed and positively regulates Hh/Gli1 signaling in mesothelioma. However, the precise mechanism needs to be elucidated. Given the emerging importance of protein kinase CK2 and Hh/Gli1 signaling in tumor initiation and progression, our findings provide important new evidence for the potential benefits of CK2 inhibitors.

## Additional file

Additional file 1: Table S1. Comparison of IHC analysis of CK2α, CK2β, Gli1 expression in mesothelioma tumors. Effects of CK2α inhibition or silencing in normal mesothelial LP9 cells. **Table S2.** Association analysis of CK2α and Gli1 expression in mesothelioma tumors (p<0.05, Chi-square). **Table S3.** Association analysis of CK2α and CK2_ expression in mesothelioma tumors (p<0.05, Chi-square). **Table S4.** Association analysis of CK2_ and Gli1 expression in mesothelioma tumors (p>0.05, Chi-square). **Figure S1.** (A) Down-regulation of two target genes of the Hh pathway (Gli1 and Ptch1) in LP9 cells, detected by quantitative RT- PCR, after CK2α siRNA treatment. (B) Cell proliferation assay after treatment with CX-4945.

## Abbreviations

CK2: Casein kinase 2 or II (inappropriate name previously used)
Hh: Hedgehog
Ptc: Patched
Smo: Smoothened
NSCLC: Non-small cell lung cancer
TBB: 4,5,6,7-tetrabromobenzotriazole
SP: Side population
ABCG2: ATP-binding cassette transporter member 2 of G family protein
