# Supplementary material for: CK2α, over-expressed in human malignant pleural mesothelioma, regulates the Hedgehog signaling pathway in mesothelioma cells
Source: J Exp Clin Cancer Res. 2014 Nov 25;33(1):93. doi: 10.1186/s13046-014-0093-6 (PMC4254219; doi:10.1186/s13046-014-0093-6)

Supplementary Table

Table 1: Comparison of IHC analysis of CK2α, CK2β, Gli1 expression in mesothelioma tumors

	CK2α -/+		CK2α ++/+++		
	Gli1 -/+	Gli1 ++/+++	Gli1 -/+	Gli1 ++/+++	total N
CK2β -/+	31.8% (7/22)	18.2% (4/22)	22.7% (5/22)	27.3% (6/22)	100% (22/22)
CK2β ++/+++	17.9% (7/39)	5.1% (2/39)	28.2% (11/39)	48.7% (19/39)	100% (39/39)

Table 2: Association analysis of CK2α and Gli1 expression in mesothelioma tumors ($p<0.05$, Chi-square)

	Gli1 -/+	Gli1 ++/+++
CK2α -/+	14	6
CK2α ++/+++	16	25

Table 3: Association analysis of CK2α and CK2β expression in mesothelioma tumors ($p<0.05$, Chi-square)

	CK2β -/+	CK2β ++/+++
CK2α -/+	11	9
CK2α ++/+++	11	30

Table 4: Association analysis of CK2β and Gli1 expression in mesothelioma tumors ($p>0.05$, Chi-square)

	CK2β -/+	CK2β ++/+++
Gli1 -/+	12	18
Gli1 ++/+++	10	21

Supplementary Figure1

A

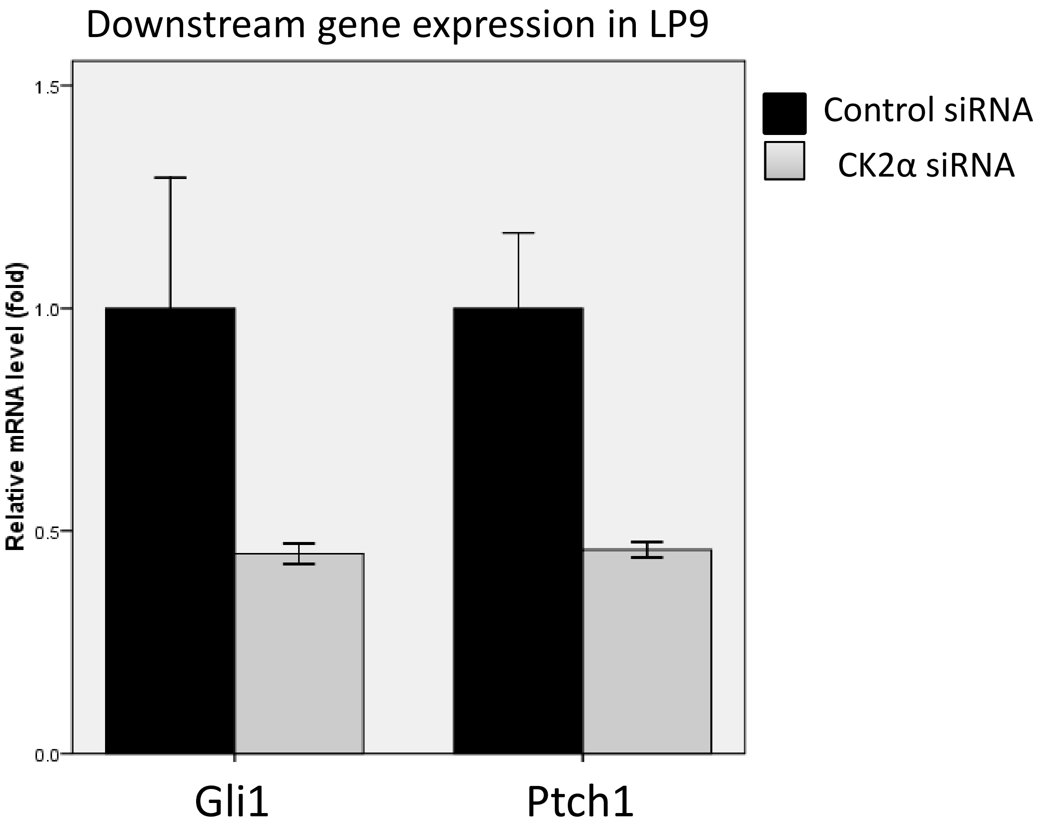

B

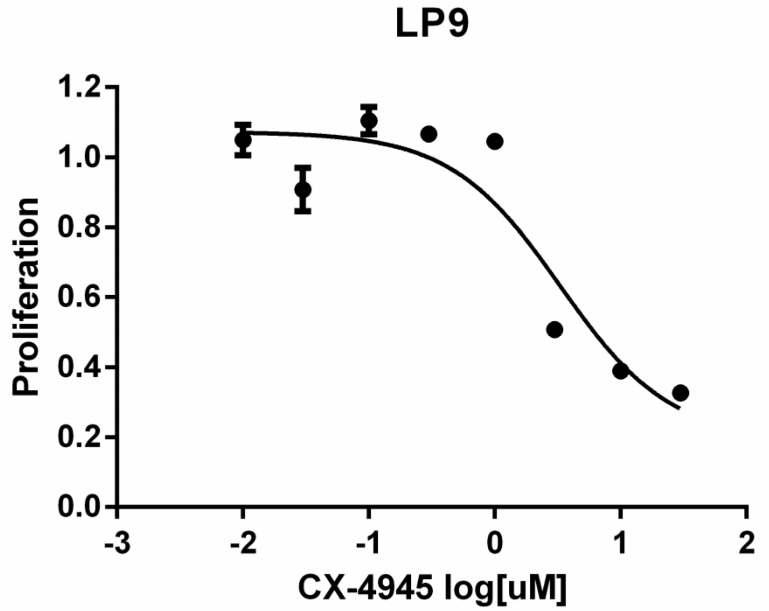

Supplement: Additional file 1: Table S1. — Comparison of IHC analysis of CK2α, CK2β, Gli1 expression in mesothelioma tumors. Effects of CK2α inhibition or silencing in normal mesothelial LP9 cells. Table S2. Association analysis of CK2α and Gli1 expression in mesothelioma tumors (p<0.05, Chi-square). Table S3. Association analysis of CK2α and CK2_ expression in mesothelioma tumors (p<0.05, Chi-square). Table S4. Association analysis of CK2_ and Gli1 expression in mesothelioma tumors (p>0.05, Chi-square). Figure S1. (A) Down-regulation of two target genes of the Hh pathway (Gli1 and Ptch1) in LP9 cells, detected by quantitative RT- PCR, after CK2α siRNA treatment. (B) Cell proliferation assay after treatment with CX-4945. [file 13046_2014_93_MOESM1_ESM.pdf]
